# Treatment Options for Acute Agitation in Psychiatric Patients: Theoretical and Empirical Evidence

**DOI:** 10.7759/cureus.6152

**Published:** 2019-11-14

**Authors:** Nicholas Zareifopoulos, George Panayiotakopoulos

**Affiliations:** 1 Psychiatry, University of Patras School of Health Sciences, Patras, GRC; 2 Internal Medicine, University of Patras School of Medicine, Patras, GRC

**Keywords:** agitation, antipsychotic, haloperidol, olanzapine, diazepam, lorazepam, aripiprazole, ziprasidone, risperidone, benzodiazepine

## Abstract

Acute agitation is a common presenting symptom in the emergency ward and is also dealt with on a routine basis in psychiatry. Usually a symptom of an underlying mental illness, it is considered urgent and immediate treatment is indicated. The practice of treating agitation on an acute care basis is also referred to as rapid tranquilization. A variety of psychotropic drugs and combinations thereof can be used. The decision is usually made based on availability and the clinician’s experience, with the typical antipsychotic haloperidol (alone or in combination with antihistaminergic and anticholinergic drugs such as promethazine), the benzodiazepines lorazepam, diazepam and midazolam as well as a variety of atypical antipsychotics being used for this purpose. Haloperidol is associated with extrapyramidal symptoms (which can be controlled by co-administration of promethazine) and may control agitation without inducing sedation, while benzodiazepines have a more pronounced sedating activity. The atypical antipsychotics aripiprazole and ziprasidone are better tolerated, while olanzapine is also a powerful sedative. Clinical trials evaluating the efficacy of different treatment options have been conducted but they are extremely heterogenous and most have numerous methodological flaws, leading to a poor overall quality of evidence upon which guidelines for the appropriate treatment could be based. The combination of haloperidol and promethazine, which combines the sedative properties of the antihistamine with the more selective calming action of haloperidol (with a reduced risk of extrapyramidal effects compared to haloperidol alone because of the anticholinergic properties of promethazine) may be the best choice based on empirical evidence.

## Introduction and background

Rapid tranquilization is a term denoting the use of pharmacological agents to calm acutely agitated or aggressive patients, both in a psychiatric as well as a general acute care setting. This review was intended to discuss exclusively the use of psychotropic drugs for the treatment of acute agitation, though other, non-pharmacological options are also available. Ever since the term was first used in the literature during the 1950s (coinciding with the advent of the first antipsychotic drugs) it has been a matter of controversy [1]. At present, it remains unclear whether the practice is ethical, legally allowed and under which conditions it is indicated. The best way to achieve rapid tranquilization has yet to be elucidated, as there are many available drugs which differ as to their exact effects, their route of administration, their duration of action as well as their side- effect profile.

Rapid tranquilization is indicated mostly for patients in acute distress who are agitated, combative or otherwise at risk for violent behavior and not amenable to reason. Under such circumstances the actions of the patients may be harmful both for themselves and others around them, so immediate interventions are warranted. These interventions however may be harmful in their own right, as they may lead to undesirable medical side effects, legal conflicts and they also may undermine the physician- patients relationship as they may also be administered without consent. The decision of whether to intervene and how is not to be taken lightly as a number of factors must be accounted for. The decision- making process, however, is further complicated by the nature of the ailment and the need for timely resolution of the situation, which necessitate that a course of action be decided upon in a matter of minutes. For these reasons, it is vital that all clinicians are well- aware of the available options, the side effects associated with each and the empirical data regarding their use in such a setting.

There are numerous options regarding rapid tranquilization and more are currently under development. The international guidelines regarding their use are conflicting at times and are being constantly updated, rendering it difficult for practicing clinicians to keep track of the matter. The purpose of this review is to examine the evidence regarding empirical options, evaluate potential novel agents suitable for rapid tranquilization and provide vital information regarding these in a manner conducive to wise clinical decision- making. The theoretical aspects of each option are provided in the first part of the review, including the purported mechanism of action of each drug, its side- effect profile and its pharmacokinetic properties. The second part of the review will examine the empirical evidence regarding the efficacy and safety of these interventions, as well as their comparison to other options, based upon the findings of clinical trials.

Treatment choice for rapid tranquilization is dependent on a number of factors, including the patient’s presentation, the availability of drugs in a certain setting and the desired effect. The therapeutic endpoint of rapid tranquilization is a matter of debate and in clinical practice, three different approaches are common [2-3]. Certain clinicians consider it best to sedate the patient completely and prefer to increase the dosage of the drug until the patient is asleep. Others may opt for lighter sedation, which retains the patient’s ability to communicate. The final approach, which is also the most suitable for contemporary clinical practice, is to administer drugs at the lowest possible dose which calms the patient and leads to resolution of behavioral symptoms, if possible without inducing sedation or sleep [2-3]. If the initial tranquilization regimen is successful, the patient can be followed up with a regular psychiatric assessment [2-3]. Accounting for the fact that not all available drugs are conducive to all of the aforementioned treatment goals, it becomes obvious that deciding upon which drug to use and at what dosage should be influenced primarily by the stated purpose of the treatment. Suitable drugs include typical antipsychotics (often co-administered with an anticholinergic agent to reduce the incidence of side effects [4], benzodiazepines and, most recently, atypical antipsychotic drugs.

## Review

No other drug has been more intimately associated in the minds of both clinicians and patients alike with rapid tranquilization than haloperidol. It is a high potency typical antipsychotic agent of the butyrophenone class, which exerts its effects primarily via antagonism of the dopamine D2 receptors. It is by no means selective and may also bind to other receptors in the central nervous system (CNS), including a1 adrenergic and sigma receptors [5]. Its effects have been thoroughly documented in the literature. It is available both in oral and parenteral formulations (designed for intramuscular (IM) administration, though intravenous (IV) administration is also possible). When administered orally, the effects are noticeable within an hour, while 10-15 minutes are required for the onset of action after IM administration. For this reason, the IM route is preferred in the setting of rapid tranquilization [6].

Haloperidol is purported to induce a state of tranquility without causing significant sedation or cognitive dysfunction, similar to other high- potency typical antipsychotic drugs. Its psychological effects are complex and, though they have been the subject of extensive study, have yet to be completely elucidated. Despite it being a first-generation antipsychotic, there are numerous advantages that favor using haloperidol instead of the newer atypical agents in certain circumstances. It is important to note that the drug has only rarely been associated with side effects other than extrapyramidal symptoms regardless of dose, and an overdose is rarely life-threatening. It has no significant hemodynamic effects, and only rarely does it affect blood pressure and myocardial contractility. Unlike other sedative drugs (including less potent antipsychotics) it does not cause respiratory depression. It may, however, prolong the QT interval, leading to life-threatening arrhythmias [7]. This effect, however, is not unique to haloperidol, and it has been more strongly associated with other antipsychotic drugs, including thioridazine, ziprasidone, and risperidone. It may lower the seizure threshold as well, though this side effect is also more intimately associated with other antipsychotics, such as clozapine, thioridazine, and olanzapine. Like all antipsychotics, its use may lead to the development of the life-threatening neuroleptic malignant syndrome, which is characterized by muscle rigidity, hyperpyrexia and altered mental status [8].

Unfortunately, haloperidol is not well-tolerated, and patients are likely to complain of a subjective feeling of dysphoria, or inner restlessness (akathisia). The drug saturates D2 receptors even at very low dosages, thus its effects are not dose-dependent [9]. However, increasing the dose seems to greatly increase the incidence of extrapyramidal side effects. The most common of these, in an acute care setting, is acute dystonia (sustained, often painful spasm of a group of muscles), which may present as an oculogyric crisis, opisthotonos or as spastic torticollis. This may occur even in the first few minutes after administration and should be promptly treated by the parenteral administration of an anticholinergic such as benztropine, diphenhydramine or promethazine [2,10]. Some clinicians routinely co-administer haloperidol with an anticholinergic in the acute care setting the prevent the incidence of dystonia. Akathisia is another severe side effect which manifests either as an inability to sit still or as a subjective feeling of restlessness. The first presentation is typical of patients who have been exposed to the drug for a few days to weeks, while the second may occur even after the administration of a single dose. It is unclear whether anticholinergics are effective for the resolution of akathisia, as it may be more likely to respond to benzodiazepines or beta-blockers [11]. In any case, the incidence of such an event is counterproductive as the purpose of the treatment is to calm, not further agitate the patient, so in the acute setting, it necessitates the immediate discontinuation of antipsychotics and the use of benzodiazepines. The duration of action is also a matter of concern, as haloperidol has a long and unpredictable half-life (ranging from 12 to 48 hours) and noticeable effects are present for at least 24 hours after the last dose. The typical dosage is 5-10 mg IM, though it was once standard practice to administer as needed (up to 60 mg per day) until sedation was achieved. Seeing as the effects are unlikely to be dose-dependent, this practice is no longer supported.

Among typical antipsychotics, haloperidol is the drug of choice in the rapid tranquilization setting. Another option is zuclopenthixol acetate, another high potency typical agent. Compared to haloperidol, it has a longer duration of action (48-72 hours) and a longer and less predictable onset. It has a more robust sedative action and is not completely devoid of hemodynamic effects, as it commonly causes hypotension [12]. Low potency typical antipsychotics such as chlorpromazine were used for rapid tranquilization in the past, but this practice is now very rare due to the side effects associated with their parenteral administration. These compounds are potent a1 antagonists and cause significant hypotension in patients who have not developed tolerance. This effect necessitated extreme caution when they were used in an acute care setting, as in certain cases they could even cause circulatory collapse. They are however less likely to cause akathisia and dystonia than haloperidol [13].

Benzodiazepines are a class of sedative drugs which have also been extensively used for the purpose of rapid tranquilization. Their mechanism of action is related to their ability to enhance the affinity of gamma-aminobutyric acid (GABA) to its GABA- A receptors, which are ligand gated chloride channels. GABA is the most abundant inhibitory neurotransmitter in the CNS and GABA-A receptors are ubiquitous, thus the CNS depressant effects of benzodiazepines are much more pronounced than those of antipsychotic compounds [14]. Certain anesthetic agents such as propofol and the barbiturates also act upon the GABA-A receptors, but their effects are more potent as they may also act as direct receptor agonists, in contrast to benzodiazepines which are strictly positive allosteric modulators at the aforementioned sites [15].

The clinical effects of benzodiazepines are typical of non- selective CNS depressants. Thus they produce notable and dose-dependent sedation, anxiolysis, sensory and motor impairment as well as anterograde amnesia, which is most often an undesirable effect though it can be beneficial in certain circumstances, most notably when such drugs are used to provide sedation during invasive medical procedures for which anesthesia is not required [16]. They also possess antiepileptic and muscle relaxant properties and are among the drugs indicated for the treatment of status epilepticus. They may cause respiratory depression in high doses, and they also act in synergy with other CNS or respiratory depressants such as opioids. Their most troublesome side- effect is the induction of a paradoxical state characterized by agitation, belligerence, and loss of social inhibitions [17]. Euphoria is not uncommon and the misuse potential of these drugs has been well documented in the literature [15]. Chronic use leads to the development of tolerance and physical dependence. In such cases, the drugs should be gradually tapered before discontinuation, as withdrawal can be life-threatening due to seizures. A specific antidote for benzodiazepines, flumazenil, is available, though its use is generally discouraged as it can precipitate seizures. In psychiatry, the main indication for their use is the short-term treatment of anxiety, insomnia and panic disorder.

The principle differentiating factors between individual benzodiazepines are their pharmacokinetic properties, namely time until onset of action, duration of action, and the presence of active metabolites. These differences also account for their differing indications: thus long-acting compounds such as clonazepam and prazepam are used mostly for the treatment of anxiety, while shorter-acting compounds such as alprazolam are preferred for panic attacks and for the induction of sleep [18]. The drugs are always administered orally in an outpatient setting.

For the purpose of tranquilizing acutely agitated patients, the drugs of choice are the benzodiazepines for which a parenteral formulation is available, namely lorazepam and diazepam. Lorazepam is generally preferred as it has a more predictable onset and duration of action, while also lacking active metabolites. It may be administered orally or IM, with the IM route typically used in a rapid tranquilization setting. Onset of action is approximately 15 minutes and the total duration of action is 8-12 hours, with 2-4 mg being a typical dose [19-20]. Diazepam may also be administered orally or IM, but IM administration has been associated with erratic absorption patterns and pain at the injection site. IV administration is also acceptable, with immediate onset and a very short duration of action (approximately 1 hour) due to the drugs' high lipid solubility which causes a redistribution of the compound from the vascular space into fatty tissue [21]. IV diazepam is among the drugs indicated for the treatment of status epilepticus [22], but it is not common in the context of rapid tranquilization. Regardless of the route of administration, the drug’s peak effects are achieved within 1 hour and residual effects last 24 hours or even longer [21].

Benzodiazepine use in the context of rapid tranquilization generally has the goal of calming the patient and ensuring adherence to follow- up treatment. Excessive sedation is generally regarded as an undesirable effect. Compared with antipsychotics such as haloperidol, benzodiazepines pose a far greater risk to the patient as they may cause respiratory depression in high doses, and may also contribute to dangerous drug interactions with other depressants which the patient may have been exposed to [23]. The risk of interactions is much greater when the patient presents to the emergency department with acute agitation, as his previous history is unknown and in many cases unobtainable until the situation is resolved, and for this reason drugs such as ketamine with a lower propensity for such interaction are preferred in the ER setting [24].

The use of a combination of a benzodiazepine such as lorazepam with an antipsychotic such as haloperidol is not uncommon in clinical practice. There is a greater risk of sedation, but the side effects associated with each drug may be mitigated in part by the other. Specifically, benzodiazepines may provide some degree of protection against extrapyramidal side effects, especially akathisia, while haloperidol may prevent the development of paradoxical agitation due to benzodiazepine use [9,25-26]. Such a combination is quite similar to the practice of neuroleptanesthesia, the combination of a CNS depressant (usually a barbiturate) with a potent neuroleptic drug for sedation during minor procedures [27-28]. This practice was common in the 1960s and 1970s but has since been abandoned, due to the development of more effective agents for anesthesia.

Haloperidol and benzodiazepines dominated the rapid tranquilization scene from the 1960s to the beginning of the present decade, when parenteral formulations of the new atypical antipsychotic drugs became available [29]. Atypical antipsychotic drugs theoretically differ from haloperidol and other typical agents due to their higher affinity for serotonin 5-HT2 receptors, and their relatively lower affinity for D2 receptors, which theoretically enables them to normalize instead of depressing dopaminergic signaling [29]. Thus, they may exert a therapeutic action in psychotic states with a relatively lower risk of extrapyramidal side effects. The aforementioned properties are shared by clozapine, olanzapine, quetiapine, ziprasidone and lurasidone [30]. Of these, only olanzapine and ziprasidone are available as parenteral formulations. Other atypical antipsychotics have a distinct pharmacodynamic profile, notably risperidone and its metabolite paliperidone which possess generally equal affinity for 5-HT2A and D2 receptors, amisulpride which is selective for the receptors D2, D3 and 5-HT7 [30], as well as aripiprazole, which is a partial agonist at D2 receptors and an antagonist at 5-HT2A. 5-HT2C and a1 adrenergic receptors [31]. An IM formulation of aripiprazole has been approved for the treatment of acute agitation.

The 3 atypical antipsychotics approved for rapid tranquilization differ greatly in regards to their side effects, overall tolerability, and duration of action. Olanzapine is a powerful sedative with its effects lasting up to 24 hours. It also enhances appetite and may cause weight gain when used for the maintenance of chronic patients. It lowers the seizure threshold and may contribute to the development of metabolic syndrome via an unknown mechanism, similar to its prototype drug clozapine. The development of EPS following olanzapine use is rare [32]. Ziprasidone has a much shorter duration of action (2-4 hours) and is generally better tolerated, as it does not cause as much sedation or weight gain. Its most alarming side effect is the prolongation of QTc interval, which may contribute to the development of fatal arrhythmias [30,33]. Aripiprazole also does not produce sedation, and notably, it is the only antipsychotic which does not cause hyperprolactinemia, indicating an effect on dopaminergic signaling profoundly different to that of other antipsychotics [31]. It has an unusual side effect profile as well, as it virtually never induces parkinsonism or dystonia, but can cause akathisia at rates similar to haloperidol. Its elimination half-life is 75 hours, leading to a long and generally unpredictable duration of action if only a single dose is used [31,34]. Another concern is that its partial agonist activity, combined with its high affinity for D2 receptors may render subsequent administration of other antipsychotics ineffective by displacing them from the receptor (Table 1).

**Table 1:** Pharmacology of Drugs Commonly Used for Acute Agitation

Table 1- Pharmacology of drugs commonly used for acute agitation						
Drug Name	Receptors affected	Biological half life	Route of administration	Main side effects	Possible life-threatening reaction	Notes
Haloperidol	D2 - D3, σ, a1	20h	Oral (tablets and oral solution), IM, (IV)	EPS	None	May be co-administered with a drug to reduce EPS, prolongs QTc
Olanzapine	D2-D3, 5-HT2A, 5-HT2C a1, H1, M1-M5	20h	Oral (regular and dipersible tablets), IM	Sedation	Respiratory Depression	
Risperidone	D2-D3, 5-HT2A, 5-HT7 a1, H1	1.5h (active metabolite paliperidone 30h)	Oral (tablets and oral solution)	Sedation, EPS	None	
Benzodiazepines (Lorazepam Diazepam Midazolam)	GABA-A (positive allosteric modulator)	Midazolam 2h, Lorazepam 10h, Diazepam 20-100h	Oral (except midazolam), IM, IV	Sedation, paradoxical reactions	Respiratory depression	May be used in combination with antipsychotics
Aripiprazole	D2-D3 (partial agonist) a1, 5-HT2A (antagonist)	75h	Oral, IM	Sedation or Akathisia	None	No EPS except akathisia, which can manifest as paradoxical worsening of agitation
Ziprasidone	5-HT2A, D2-D3, 5-HT1 (partial agonist)	7h	Oral, IM	Minor sedation, usually well tolerated	Cardiac arrhythmias	Prolongs QTc
Droperidol	D2-D3	2h	IM, IV	EPS	Cardiac arrhythmias	Also used in anesthesia, prolongs QTc
5-HT- 5-hydroxytryptamine/serotonin, a1 – alpha 1 adrenergic receptors, D – Dopamine, EPS- Extrapyramidal symptoms, GABA- Gamma-Aminobutyric acid, H1- Histamine Receptor 1, IM-Intramuscular, IV-Intravenous, QTc- Corrected QT interval in electrocardiography. QT prolongation is associated with the development of life-threatening ventricular arrhythmias (Torsades des pointes)						

Quetiapine has a similar pharmacological profile, though its affinity to D2 receptors is lower and thus it is even less likely to induce akathisia or extrapyramidal symptoms [35]. Due to its profound sedative properties especially in an acute setting in individuals without tolerance, it may be used off-label to calm agitated individuals, a practice which is quite common in Greece, albeit more so in the internal medicine inpatient wards compared to the emergency department or the inpatient psychiatry wards [36]. Furthermore, psychiatric patients who have been exposed to quetiapine or similar atypical agents may be resistant to the sedative effect, and it has also been associated with paradoxical agitation or even manic reactions [37-38]. For these reasons and because no parenteral formulation is available, it is not considered a viable alternative to atypical agents mentioned above for acute agitation in psychotic patients. It has been evaluated for the treatment of agitation in patients with delirium or dementia, though this practice remains controversial [39-40].

It becomes clear that each treatment option for the attainment of rapid tranquilization has a unique effect profile, rendering it difficult to decide which drug is best suitable for a particular case. As is almost always the case when prescribing psychotropic drugs, treatment must be individualized and adjusted as needed until the needs of the patient are met, with the choice influenced heavily not only by the expected beneficial effects of the drug, but also by its side effect profile. While the clinical and subjective acute effects of both haloperidol and the benzodiazepines have been extensively documented in the literature, the same is not true regarding the new atypical antipsychotic drugs, though this is likely to change as they are more extensively utilized in the acute care setting. For the second part of our study, we opted to review the empirical evidence regarding the efficacy and risks of each of the aforementioned drugs, based on randomized controlled trials evaluating them in a rapid tranquilization context.

From the data presented in the previous sections, it seems quite clear that a drug of choice for rapid tranquilization cannot be decided upon on a purely theoretical basis. Empirical evidence evaluating the use of these drugs in a clinical setting and comparing them against each other would enable a final conclusion to be drawn. Numerous clinical trials have been conducted, utilizing both the oldest available drugs (haloperidol and benzodiazepines) as well as the more recent ones (atypical antipsychotics) [34,41-43]. Comprehensive systematic reviews are available for benzodiazepines [23] (including trials of different drugs of this class), haloperidol [10], droperidol (a haloperidol analog with a shorter duration of action used mostly in anesthesia) [44], the combination of haloperidol and promethazine [4] and the atypical antipsychotics olanzapine [45], risperidone [46] and aripiprazole [47]. Clinical trials involving other drugs, including ziprasidone have been published, but systematic reviews of these trials are not currently available.

The heterogeneity of the literature seems to preclude a comprehensive systematic review evaluating the empirical evidence pertinent to all the available drugs. Shortcomings of the literature include a small sample, different inclusion and exclusion criteria, insufficient blinding, non-standardized outcome measures, non- uniformity of dosages among different studies and a trial setting that is in most, if not all cases, not generalizable to clinical practice. Certain common practices such as administration of haloperidol as needed, drug polypharmacy and extremely high doses of haloperidol have never been evaluated in a controlled setting. The risks of such an approach almost certainly outweigh the benefits, even if life-threatening adverse reactions are rare.

In clinical trials of rapid tranquilization protocols, it is uncommon to use a placebo as a comparator, as such an approach may unnecessarily endanger the patients enrolled in the control group. That said certain studies did compare the active drug with placebo [48-50], though they were in the minority.

Thus, the regimen to be evaluated is usually compared to the standard of care, which is 2.5-5 mg of haloperidol IM. This explains in part the fact that haloperidol appears in almost every trial regarding rapid tranquilization. Haloperidol is also an old drug, readily available in almost every medical center in the world, reliable and quite cheap in comparison to more recent drugs. Since it can most likely be used even when no other drugs are available, it makes sense for it to be a reference point for the empirical studies on rapid tranquilization. This, however, leaves the question of whether the more recent drugs are in any way superior to it unanswered, as trials including those drugs are comparatively fewer, though better designed [10,46]. The combination of haloperidol and promethazine may be pharmacodynamically beneficial, as promethazine has a sedative effect which may synergize with haloperidol and also possesses anticholinergic properties which confer a certain degree of protection against extrapyramidal side effects [4].

It is not possible to infer from recent studies whether any of the novel drugs is superior to haloperidol (or whether they differ substantially from each other, for that matter). The most common finding is that all drugs used in practice for rapid tranquilization do, in fact, exert a calming effect and reduce agitation, and aggression, while being associated with significant side effects. Haloperidol (and the associated droperidol) is most likely to cause extrapyramidal effects, while olanzapine and the benzodiazepines are associated with greater sedation (Table 2).

**Table 2:** Empirical Evidence on Commonly Used Treatment Choices for Acute Agitation

Table 2- Empirical Evidence on commonly used treatment choices for acute agitation								
Treatment Regimen	Availability of Systematic Review	Number of Randomised clinical trials with comparison group	Total number of patients	Main Outcomes Measured	Direct comparisons	Conflicts of Interest in published clinical trials	Main conclusion of review	Quality of evidence
Risperidone	Yes [46]	9	582	Need for restraints, Positive and Negative Syndrome Scale - Psychotic Agitation Sub-score (PANSS-PAS), Modified Overt Aggression Scale (MOAS)	Quetiapine Haloperidol, Olanzapine, combinations of risperidone with Valproate and oxcarbazepine	No	Risperidone does not seem superior to any of the comparators,	Very Poor
Aripiprazole	Yes [47]	3	885	Agitation at 2 hours, need for additional treatment	Placebo, Olanzapine, Haloperidol	Yes	Aripiprazole may be effective for the treatment of acute agitation, but the limited number of studies diminishes the strength of the evidence	Very Poor
Haloperidol	Yes [10], renewed periodically	41	4933	Asleep by 30 minutes, Need for additional medication	24 regimens in total, including all drugs of the table, zuclopenthixol, chlorpromazine, olanzapine, ziprasidone and many benzodiazepines	No	Haloperidol is effective and relatively safe for treating acute agitation, though it is associated with side effects. In many cases, it may be the only suitable drug available	Poor
Droperidol	Yes [44]	4	857	Asleep at 30 minutes, Need for additional treatment	Haloperidol, Placebo, olanzapine, midazolam	No	Droperidol seems effective and better tolerated than comparators for the treatment of acute agitation	Good
Haloperidol plus Promethazine	Yes [4]	6	1367	Asleep at 30 minutes, need for additional treatment, need for restraints	Haloperidol alone, Ziprasidone, midazolam, lorazepam, olanzapine,	No	Haloperidol plus promethazine is superior to Haloperidol alone for treatment of acute agitation, and less sedating than benzodiazepines	Good
Olanzapine	Yes [45]	13	2031	PANSS-EC, need for additional treatment, asleep at 30 minutes	Haloperidol, Lorazepam, placebo, haloperidol and promethazine, Ziprasidone	Yes	May be more effective and better tolerated than haloperidol alone, but data is not sufficient to evaluate against other comparators	Poor
Benzodiazepines with or without haloperidol	Yes [23]	21	1968	Global Impression- Improvement, Need for additional treatment	Placebo, Haloperidol alone, Olanzapine, Ziprasidone	No	Benzodiazepines are more sedating than antipsychotics bau less effective for the management of acute agitation, while the combination of benzodiazepines and antipsychotics leads to more side effects without conferring additional efficacy	Very poor

Benzodiazepines may be less effective than neuroleptics, a finding which seems plausible, as they do not address the underlying psychopathology and they also do not cause significant sedation at the dosage range most commonly employed in practice. Ziprasidone, aripiprazole, and risperidone may be better tolerated in the short term, but the relevant studies to support such a claim are far fewer than the studies for the aforementioned drugs.

## Conclusions

In conclusion, it is worth noting that the right choice of drug for rapid tranquilization remains a matter of clinical judgement until studies with a larger sample are conducted in settings which are more similar to actual practice conditions. Olanzapine has numerous advantageous properties, as its most significant short-term effect is excessive sedation which is actually desired in this context, and it not prone to cause akathisia or extrapyramidal symptoms which can worsen agitation. However, it is expensive and IM formulation are not always available. Haloperidol, alone or in combination with an antihistamine-anticholinergic is another viable candidate due to the comprehensive evidence base and the decades of clinical experience in favor of its efficacy, while recent findings are also in favor of a combination of haloperidol and promethazine. In many cases haloperidol (alone or in combination with an anticholinergic) is, in fact, the only choice. Benzodiazepines should not be used as monotherapy, though adding a low dose to a neuroleptic regimen makes sense for patients with a history of anxiety or a seizure disorder. Droperidol, ziprasidone, aripiprazole, and risperidone may be seen as second-line drugs even in settings where they are available, as neither empirical evidence nor a theoretical framework exists to justify a preference for using them instead of first-line drugs. Based upon the available evidence, we would primarily prefer to use olanzapine (if available) in most patients without significant medical comorbidities and a combination of haloperidol and an anticholinergic in all other cases.
